# Exploring the features of mobile phone application of anatomy in basic medical sciences: a qualitative study

**DOI:** 10.1186/s12909-020-02145-x

**Published:** 2020-07-20

**Authors:** Mahmoud Mansouri, Shoaleh Bigdeli, Afsaneh Dehnad, Zohreh Sohrabi, Somayeh Alizadeh, Mohammad Hasan Keshavarzi

**Affiliations:** 1 Center for Educational Research in Medical Sciences (CERMS), Department of Medical Education, School of Medicine, Iran University of Medical Sciences (IUMS), Tehran, Iran; 2 Department of English Language, School of Health Management and Information Sciences, Tehran, Iran; 3 Clinical Education Research Center, Shiraz University of Medical Sciences (SUMS), Shiraz, Iran

**Keywords:** Feature, Anatomy, Mobile phone, Application, Educational technology

## Abstract

**Background:**

The importance of mobile phones has become one of the new research topics in health professions education due to the ease of access and flexibility. Although novel approaches to health professions education recommend the use of educational technologies, such as mobile applications, a limited number of studies have been conducted with regard to learning anatomy through mobile applications. Considering the increasing needs of medical students for mobile technology to meet their educational needs, wants and desires, we decided to explore the features of an anatomy mobile application.

**Methods:**

This qualitative study was conducted in two stages of holding focus groups, and an expert panel session. Students of basic Medical sciences, and faculty members of anatomy at Iran University of Medical Sciences formed the research participants. Semi-structured interviews and note-taking were used to collect the data. Moreover, Brown and Clark methods were used for thematic analysis. Finally, four criteria presented by Lincoln and Guba for qualitative studies were used to ensure the credibility, confirmability, trustworthiness and transferability of the data.

**Results:**

Based on the data analysis, 37 codes that could be used to design anatomy mobile content for medical students were extracted. These features were categorized into eight main themes of “visual richness”, “scientific comprehensiveness”, “auditory richness”, “affordability”, “user-friendliness”, “self-assessment”, “interactive content” and “user support”.

**Conclusion:**

This study explored the features of an anatomy application that can be used by educational app developers. Anatomy departments at Medical Universities, policymakers, and curriculum planners in the field of medical education can also adopt the findings of the present study.

## Background

The world has recently witnessed drastic changes in teaching methods which pose unavoidable challenges to traditional education systems. Information and communication technology, as a novel phenomenon in the world today, has deeply altered various aspects of education in general and teaching and learning process in particular [1, 2]. In traditional education systems, the main approach to teaching was lectured-based teaching, while currently, with the emergence of novel educational technologies, and the new trend emphasizing on knowledge enhancement, medical students can benefit from new approaches to learning, which have received increasing attention over time [3]. Nowadays, the use of mobile tools, and more specifically, mobile phones which are easily accessible to everyone, provides a valuable opportunity for teachers to facilitate the teaching-learning process, offer equal educational opportunities, and improve teacher-learner communication [4]. Although some believe that the use of mobile phones for learning may cause distraction, ending up using social media or playing games, and that the small size of the screen may cause difficulty in displaying the total information needed at a time [5, 6], it has become a major topic of education and research in medical education due to ease of accessibility at anytime and anywhere [7]. Mobile phones are considered to be appropriate for enhancing the quality of medical education, and this is the reason for the high popularity of mobile phones as effective tools for learning [8]. They reduce the workload of in-person education system, provide a round-the-clock learning experience, facilitate education, and reduce educational costs [3]. It has also been reported that the use of mobile phones improves patient care, promotes the accuracy of diagnosis, and saves time [9]. With regard to the pressure of modern trends on higher education, and based on the positive attitudes and perceptions of students, mobile apps are important and effective novel means for educational purposes [10].

Anatomy as a basic course for medical students includes different sections with several components. The content of the course focuses on the structure of and the relationship between different parts of the human body [11]. Learning a large amount of materials is highly demanding for the students since the topics are complicated with a large amount of details to learn and memorize. Therefore, many students try to employ tools other than print resources to learn and pass their exams. These tools are typically based on modern educational technologies and include images and text which could be depicted in smart phones.

In anatomy classes in Iran, a given topic is first introduced through a lecture with the aid of slides or a short video clip. Next, the topic is more elaborated through small group learning method with the supervision of an instructor. Then, the students work on anatomical moulage, and virtual dissection table. Finally, they work on a cadaver. Medical students in Iran, as reported in the focus group, mostly use Netter’s Atlas of Human Anatomy [12], which fails to meet all their needs and preferences. For example, although the description of cross sectional anatomy is not the primary focus of the atlas, the frontal and sagittal planes are explained in more detail while transverse or rotational planes are relatively less explained. Moreover, the Atlas lacks a search section and it takes time to find some details, as stated by some students. In some sections, the explanations and labels are not complete, and the students have to refer to other sources or pay to obtain necessary information. Another problem is that the video clips are not free and should be purchased. Finally, there are some parts in the curriculum developed by the anatomy department of the university but are not covered in Atlas.

The development of a mobile app on the basis of the local educational needs for learning anatomy would increase students’ interactions, and motivation to improve their learning, and engage them in the process of learning more effectively [13]. The use of mobile phone technology can provide new opportunities for the improvement of learning anatomy [14]. The findings of a recent study suggest that learners who use mobile phones for learning anatomy, compared to those who have access to teachers and print sources, are more successful in achieving the specific learning objectives [14]. However, to the best knowledge of the researchers, there are a few studies on the features of a mobile application for learning anatomy.

The current approach to medical education recommends, the use of educational technologies, including mobile phones because of the potential convenience, and ease of access of these tools [15]. However, due to the scarcity of studies on learning anatomy through mobile apps, and drawbacks of the available anatomy app, Netter’s Atlas, the present study was conducted to explore the features necessary for developing a mobile app for undergraduate medical students. The findings could contribute to the development of an anatomy mobile application based on learners’ expectations and teachers’ perceived educational needs. In particular, the present study was performed to identify the features which are required for developing a mobile app of learning theoretical anatomy for undergraduate medical students with a focus on learners’ expectations and teachers’ perceived educational needs.

## Methods

This qualitative study was conducted at the school of medicine, Iran University of Medical Sciences (IUMS) in 2019. A purposive sampling was carried out from the population of undergraduate medical students, and anatomy faculty members.

It was decided to have Focus Group (FG) and Expert Panel (EP) sessions to collect data for mobile apps features which could be deployed for designing and developing such an app in the future. FG is typically performed because the participants have similar knowledge and ideas on a given topic. FG is believed to provide richer information than individual interview due to its group dynamics and interactions among participants. It shows how the participants feel about or behave towards a topic, and is recommended for generating ideas. It could be used alone or accompanied with other techniques [16–18]. In addition to FGs, it was decided to have an EP session with anatomy faculty members to evaluate some generated ideas, discuss the topics, and come to a conclusion or recommendations [19, 20].

The first FG session was held upon obtaining the student consent form. The participants consisted of nine sixth semester medical students, who had passed anatomy courses (both theoretical and practical parts) and were familiar with the topics. In the first FG session, a list of their expectations was developed. However, to receive more information and to confirm some data, another FG session was held with eight participants. On the whole, there were 17 participants, 10 males and 7 females, (21–22 yrs.). For the inclusion and exclusion criteria see Table 1. Both FG sessions, which took about 45–55 min were held at the office of Student Research Committee. Three members of the research team directed the sessions. One researcher (MM) who was a masters student of medical education at the time, worked as the moderator. He explained the objectives of the study, the purpose of holding the session, and that their points of view, as the end users, were important. The prompts were selected by literature review and were checked by the research team prior to the FG sessions. In particular, the questions were based on the study by Harmon (2015), whose research design was very similar to that of our study [21]. The participants were asked questions about the drawbacks and advantages of Netter’s, the specific features of an application they would like to have on their mobile phones, the topics to be covered, and the features which needed to be improved in the upcoming application as compared with Netter’s. The moderator (MM) also asked probing questions, such as “Could you explain this more?” or final questions such as “Do you have anything else to add?” to improve the discussion and the data collection process. Another researcher (MK) who was the first facilitator, was a PhD graduate of medical education. He had access to the framework of the questions, and monitored the accuracy of data collection. The third researcher (SA), a PhD student of medical education at that time, was responsible for taking notes. A digital voice recorder was used to record the discussions which were then transcribed verbatim for analysis.

**Table 1:** Inclusion and Exclusion Criteria for the FGs

**Inclusion Criteria**	Being undergraduate medical students studying at Iran University of Medical Sciences
	Having passed the anatomy course
	Studying at the 6th or 7th semester of undergraduate medical program
**Exclusion Criteria**	Being guest or foreign student
	Having started clinical courses
	Currently being enrolled in the anatomy course

The trustworthiness of this study was supported by four criteria: credibility, dependability, confirmability, and transferability proposed by Lincoln and Guba [22]. We recorded the participants’ voice and reported their statements in quotations. We also had a member check and asked the participants to verify their statements. The data coding was discussed and performed by the team of researchers. Moreover, we reported the participants’ characteristics and described the study procedure in detail for transferability purposes.

The data resulting from the FG sessions were analyzed by using thematic analysis as the most common technique for data analysis [18], based on the six-step model proposed by Clarke and Braun 2006 [23]. Thematic analysis is an inductive technique moving from the parts to the whole, through reflecting upon the data, provided by the research participants whose comments are divided into smaller parts to extract themes. To become familiar with the data, on the basis of Clarke and Braun’s model, the researchers read and re-read the data several times to find the preliminary ideas, and extract codes in the next steps of analysis. Afterwards, the codes were categorized, and then primary or secondary themes were specified for each category. Ultimately, the final or major themes were extracted and defined. Thereafter, the final report was tabulated. For the data analysis, several meetings were held with the research team, including the supervisor (AD), advisors (SB) and (ZS), mediator (MM), and one of the facilitators (SA). In each meeting, the problems were discussed and resolved, the differences were reconciled, and the decisions were made to assist accurate analysis. However, there were some ambiguous points made by the participants. Therefore, member check was performed; the participants were contacted individually through phone calls, social networks, or in person and were requested to explain some confusing points. In the re-check part, the complementary and clear explanations of the participants on ambiguous points were provided.

**Fig. 1:**
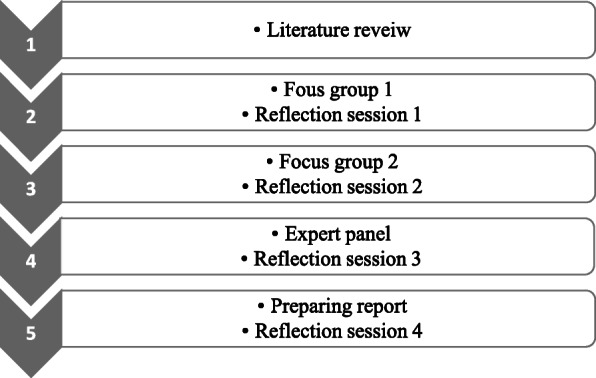
The process of conducting the research

In the next step, an EP was held with five anatomy faculty members. The supervisor (AD), advisors of the project, a software engineer, the facilitators (SA) and (MK), and the mediator (MM) were present in the session. A summary of the topics was discussed and the goals were made clear to the participants. Moreover, the process of the research was explained, and the report of the FG recorded by the team was presented. Each members of the EP was asked to discuss the themes identified, and comment on them. Then, a polling of agreement was performed by vote on approving or disapproving the items. The discussions were recorded by using a voice recorder and then transcribed verbatim. Furthermore, the facilitator (SA) took notes. The other facilitator (MK) was present for polling the votes. The items were presented by the mediator (MM). If there were any items which needed technical advice for approval, the softer engineer would comment on them. The session took about 50 min, and was held at the department of medical education in the medical school of IUMS. Figure 1 summarizes the process of conducting the research.

## Results

In the analysis of the FG data, 121 ideas or preliminary codes were initially obtained. Then, by eliminating similar, overlapping, or repeated codes, 30 codes were specified. The codes were compared and categorized on the basis of their similarities and differences, resulting in 11 sub-themes which were finally classified under 8 themes. These themes were then discussed in the EP. Analyzing the data of the FG, we were able to identify some codes denoting the same topic; the similar codes were then assigned to one sub-theme.

Visual features of the app were very important to the participants; for example, Participant 7 said: “Being three-dimensional makes it great to see all the parts we want”. Similar codes focusing on visual features were titled “appropriate visual design” as a relevant subtheme. Other codes, related to another category, were allocated to the sub-theme “educational clips”. Eventually, upon further analysis of the codes and sub-themes, it was decided to classify these two sub-themes under a general theme titled “visual richness”.

Other features mentioned by the students included: “the source of the scientific content”, “the extent to which the details are presented”, and “how comprehensive the functions are shown”. Here is what participant 10 said:

*It should be based on presenting, organs, systems, and region, or if it is made for the students of a university, it will be based on the educational programs. It's much better if it's based on the topics and the lessons that are offered by the anatomy departments.*

Two codes emerged from students’ emphasis on curriculum, and were assigned to the sub-theme “credible materials and curriculum”. Some other related codes were assigned to the sub-theme “provision of comprehensive scientific content”, as well. Then, it was found that the two sub-themes were similar and could be allocated to the same category; thus, these sub-themes, due to their similarities, formed the theme “scientific comprehensiveness”. The students had different ideas about the auditory feature of the application. Here are three excerpts:

*It should only be in the form of pronunciation, so that we can understand the correct pronunciation of units, such as nerves or muscles. [Participant 6]*

*In general, if the pronunciation of the units is given it is excellent. [Participant 9]*

*If possible, the sounds are in the form of a link on a separate site so that someone can choose, especially sounds that have long descriptions*. [Participant 4]

The codes related to the topic of “sound” were allocated to the sub-theme “optional audio feature”. By reviewing the data and the sub-themes, the final theme called “auditory richness” was formed.

Another code belonging to a separate category, “reasonable price”, was put under the sub-theme “economical”, which was later changed to “affordability”, based on the students’ statement about the reasonable price of the application. Here is what Participant 1 said:

*The price of the program is very important to be reasonable.*

There were other related codes which were categorized under the theme “user-friendliness”. Moreover, similar codes were categorized under the sub-theme “relevant tests”. Eventually, this category was assigned to the theme “self- assessment”. Some other codes belonging to the same category were titled “interactive instructional design” which was then changed to “interactive content”. There were two other related codes, which were classified as the sub-theme “having a support/guide option” for the theme “user support”. The results are shown in Table 2. The indicators or students’ quotations are available on request.

**Table 2:** Codes, sub-themes, and themes extracted from the FG

Codes	Sub-themes	Final theme
Being three-dimensional (3D) Containing real and appropriate images Having sufficient and appropriate graphics Having an appropriate visual design Having animation	**Appropriate visual design**	**Visual richness**
Having educational clips	**Educational clip**	
Being based on a credible sources Compatibility with accredited curricula and syllabi	**Accredited curriculum**	**Scientific comprehensiveness**
Offering important contents such as diseases and their complications Containing features and details Being classified on the basis of regions and systems Showing functions Having comprehensive and reliable source	**Comprehensive content**	
Not having disturbing sound Having a loud enough sound Offering the pronunciations of words	**Optional audio feature**	**Auditory richness**
Having a low price Having a reasonable price Requiring low payment for subscription	**Economical**	**Affordability**
Having a reasonable file size Not being time-consuming Being easy to work with Not having unnecessary content Providing written explanations	**Appropriate content** **design**	**User-friendliness**
Having a note-taking feature and allowing to save certain items	**Customization**	
Containing tests for different levels	**Relevant tests**	**Self-assessment**
Having a zoom option Separating important parts and being responsive Having a search option and being interactive	**Interactive instructional design**	**Interactive content**
Supporting the user in understanding the course content No support is required	**Support and help option**	**User support**

As for the EP, the recorded session was listened to, and reviewed several times to facilitate the transcription process and to have a good understanding of the content of the codes. After the transcription process, the data were compared with the notes taken during the interviews, and both were examined carefully. Although the EP initially discussed some codes, they eventually confirmed all the codes extracted from the FG. However, they added some new codes (seven codes) to the previous ones (See Table 3). In this way, while assessing the learners’ expectations, the EP helped us to make a better understanding about the features of a mobile anatomy app. These codes were discussed, reviewed and analyzed by the team of researchers.

**Table 3:** Learners’ codes and expert panel’s new codes

Learners’ codes	New codes added and codes confirmed by the expert panel
Being three-dimensional (3D) Containing real and appropriate images Having sufficient and appropriate graphics Having an appropriate visual design Having animation	“...using sample image of cadaver”.
Having educational clip	Confirmed
Being based on credible sources Compatibility with accredited curricula and syllabi	“....being based on a specified building blocks of anatomy”
Offering important points such as diseases and their complications Containing features and details Being classified based on regions and systems Showing functions	“....moving from surface to depth” and “displaying adjacencies”
Not having disturbing sound Having a loud enough sound. Offering the pronunciations of words	Confirmed
Having a low price Having a reasonable price	Confirmed
Having a reasonable file size Not being time-consuming Being easy to work with Not having unnecessary content Providing written explanation	“…. having labeling option” “…. having language option” “not looking cluttered”
Having a note-taking feature Allowing to save certain items	Confirmed
Containing tests Having tests for different levels	Confirmed
Having a zoom option Separating important parts Having a search option	Confirmed
Supporting the user in understanding the course content No support is required	Confirmed

Here are what two members of the panel stated:

*It must have an image or clip on cadaver, so the students could examine and study the parts on their own*. [A]

*There are a few number of cadavers which should be examined under the supervision of an instructor; but if the image is available on the application, they could examine it independently*. [C]

On the basis of these indicators, a new code was formed, and we agreed it could be placed under the theme “visual richness”.

They also discussed the content and topics which should be based on recent curriculum. Participants E and C said:

*It is better to consider one block for each section because each block requires a large volume of content*. [E]

*It is much better to present parts from the surface to the depth*. [C]

As what they discussed and agreed on was based on the recent curriculum of medical school, we decided to categorize it under the theme of “scientific comprehensiveness”. They all agreed that an anatomy application should have labeling and language options which could be the same as “user-friendliness”.

Regarding the rest of the codes, the members of the panel agreed with the codes extracted from students’ statements.

**Fig. 2:**
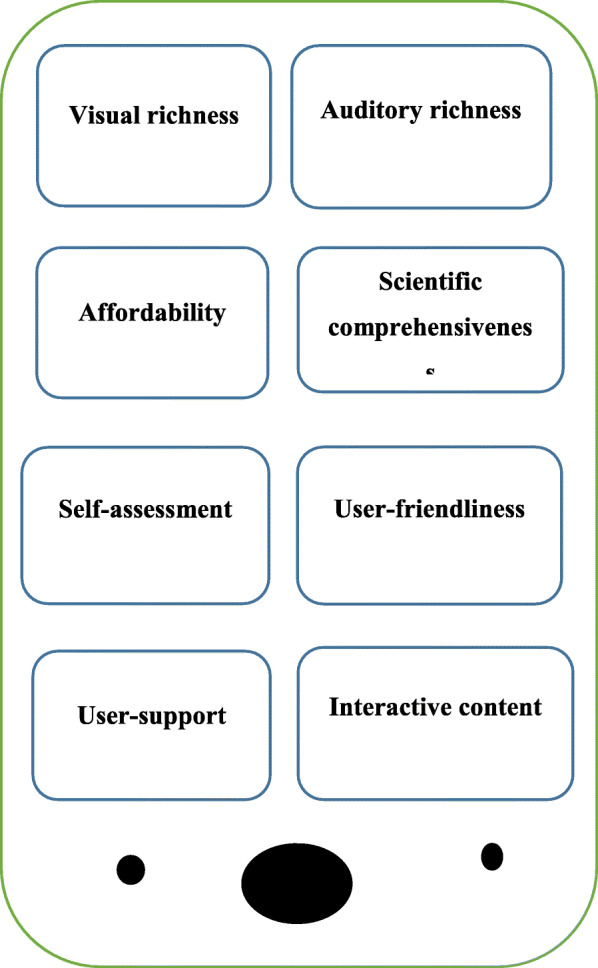
Major themes for developing a mobile application of anatomy

Evaluating the codes extracted from the FG sessions, the EP confirmed them all, and added seven new codes: “using sample image of cadaver”, “... being based on specified building blocks of anatomy”, “... moving from surface to depth”, “displaying adjacencies”, “… having labeling option”, “having language option”, and “not looking cluttered”. Upon specifying the highlighted, similar, and frequent features, based on the data obtained from the FGs and EP, the researchers extracted the features of a mobile app for learning anatomy (Fig. 2).

## Discussion

The present study aimed to shed light on the features of a mobile app for learning theoretical anatomy course of the undergraduate medical curriculum. The following themes were extracted for developing such an application: “visual richness”, “scientific comprehensiveness”, “auditory richness”, “affordability”, “user-friendliness”, “self-assessment”, “interactive content”, and a “support option”. Based on the results, visual richness is a major theme with two sub-themes of “appropriate visual design” and “educational clip”. The visual properties of the app, is very important and will enhance its effectiveness, especially for the course of anatomy because it is mostly visual. This is in line with the result of other studies which showed that the use of appropriate images and educational clips are essential for mobile learning [21, 24–26]. The importance of the visual features of apps has also been highlighted in the study by Crook et al. (2017) who conducted their studies through FG sessions [27]. However, contrary to the findings of other studies, the participants of this study preferred real images of body organs and even images of cadavers.

According to the results, “scientific comprehensiveness” is another important theme with two sub-themes of “using credible content, accredited curriculum and syllabi”, and “presenting a comprehensive scientific content”, indicating that the educational content used in apps must be reliable, otherwise it is not appealing in the process of learning. The results are consistent with those of the study by Matheus et al. (2016). They investigated the status of mobile apps in terms of health-related behaviors and used a framework known as Persuasive Systems Design model to evaluate the features of mobile apps. Consistent with our results, features such as “reliability” and “credibility of the content” were extracted in that study [28]. By referring to accredited curricula and syllabi, the participants of our study meant the prescriptive curriculum of anatomy approved by Iran Ministry of Health and Education, and the syllabi developed by the anatomy department of the university. This emphasis on a local centralized curriculum and syllabi has not been observed in other studies.

Another theme extracted in the present study was “auditory richness”. Considering this theme ensures that the app is rich in terms of sound features. This theme contained a sub-theme entitled “having a sound option”, with three components: “having a loud enough sound”, “not having sound”, and “offering the pronunciations of words”. It means that learners can use the content of the app based on their own preferences. The findings are in agreement with those of the study by Liao et al. (2017) who investigated the essential and attractive functions used for promoting sports skills in mobile apps and found that the features of such apps could influence the quality of the users’ perception [24]. However, our participants preferred the sound to be optional, and our expert panel suggested the “language options”. The option of “language” was not reported in other related studies.

The theme “affordability” consisted of a sub-theme of “being economical”, i.e., having an appropriate, low or reasonable price. This sub-theme indicates that the app must be affordable for the users so that it can be easily accessed by all or most of the learners, because students, who are the main users of this app, do not have an income. This finding is consistent with that of the study by Nitsch et al. (2016) who found affordability as an important feature of the apps [29]. However, affordability is very important for the medical students in Iran since anatomy apps especially those with comprehensive information and images are more expensive than other applications.

Based on our findings, another theme is “user-friendliness”. The codes belonging to the sub-themes “customization and” and “content design” include: “having a reasonable file size”, “not being time-consuming”, “ease of use”, “containing necessary content”, “providing written explanations”, “having a labeling option”, “having language options”, and “not looking cluttered”. User-friendliness means that everything should be designed on the basis of the end users’ preferences, i.e., the students. This will result in an acceptable app and promote learning. The results of our study are in accord with those of the study conducted by Zilverschoon et al. (2019). They compared the features of electronic apps for teaching 3D anatomy to provide a guideline for the selection of appropriate apps. They found non-commercial apps to be promising in terms of ease of use [30].

The next theme is “self-assessment”. When using this app, the users should be able to assess their own strength and weaknesses and improve their performance. A test or quiz provided at the end of each topic can help learners assess themselves and resolve their weak points. This finding is consistent with the results reported by Chi Yan Hui et al. (2017) who examined the use of mobile apps for helping self-management in patients with asthma. The study was conducted to identify the features related to clinical effectiveness. The data analysis revealed several features, including the features of showing questions and feedback [31].

Another theme was “having an interactive content”. The “zoomability”, “separating different parts”, and “having search option” are the related items in this category. These findings are also emphasized in the study by Louise et al. (2014) who worked on complementing the teaching of anatomy by using an anatomy app. They reviewed 27 anatomy apps and found that features such as “having a zoomability on each anatomical structure” and “ease of separating a structure”, and “explaining each part separately” were positive features highlighted in some popular apps [26].

Based on the findings, “user support” is another theme with the sub-theme of “having a support” or “help option”. The existence of a support or guide in the app allows the users to resolve the problems they encounter while using it. However, few students mentioned that it was not necessary to have such a support, because in the case of an ambiguity, one can easily find the answer from the Internet. Similarly, Crook et al. (2017) investigated the interface of a mobile app for individuals with communication disorders and found that the existence of ready messages for support was a major issue in apps which deserved attention [27].

## Conclusions

The findings of the study revealed eight features including “visual richness”, “scientific comprehensiveness”, “auditory richness”, “affordability”, “user-centeredness”, “self-assessment”, “interactive content”, and a “user support”. These findings can assist educational mobile app developers to design anatomy or similar apps in the field of medicine. In addition, departments of anatomy can benefit from the findings of this study to be aware of students’ needs and expectations in anatomy courses. The findings can also help policy-makers and curriculum developers in general, and policy-makers in the domain of e-learning and mobile learning in particular, become familiar with the students’ expectations, and their educational needs, and preferences. Another study, in which participants from other universities are included, is suggested to increase the generalizability of the findings. It is also recommended that a survey study be conducted on the basis of the findings of this study.

## Data Availability

The data and materials used in this study are available upon the request.

## Abbreviations

IUMS: Iran University of Medical Sciences
FG: Focus Group
EP: Expert panel
